# Determinants and Effects of Vitamin D Supplementation in Postmenopausal Women: A Systematic Review

**DOI:** 10.3390/nu15030685

**Published:** 2023-01-29

**Authors:** Mohammed M. Hassanein, Hasniza Zaman Huri, Kauser Baig, Abduelmula R. Abduelkarem

**Affiliations:** 1 Department of Clinical Pharmacy and Pharmacy Practice, Faculty of Pharmacy, Universiti Malaya, Kuala Lumpur 50603, Malaysia; 2 Department of Obstetrics and Gynecology, University Hospital Sharjah, Sharjah P.O. Box 27272, United Arab Emirates; 3 Department of Pharmacy Practice and Pharmacotherapeutics, College of Pharmacy, University of Sharjah, University City Road—University City, Sharjah P.O. Box 27272, United Arab Emirates

**Keywords:** menopause, vitamin D status, 25-hydroxyvitamin, 1,25(OH)D, vitamin D deficiency, nutrients

## Abstract

Hormonal fluctuations, excessive clothing covering, sunscreen use, changes in body fat composition, a vitamin D-deficient diet, and a sedentary lifestyle can all predispose postmenopausal women to vitamin D deficiency. An effective supplementation plan requires a thorough understanding of underlying factors to achieve the desired therapeutic concentrations. The objective of this study was to conduct a systematic review of the predictors that affect vitamin D status in postmenopausal women. From inception to October 2022, we searched MEDLINE, Embase, Web of Science, Scopus, and clinical trial registries. Randomized clinical trials of postmenopausal women taking supplements of vitamin D with serum 25-hydroxyvitamin D (25(OH)D) measurement as the trial outcome were included. Two independent reviewers screened selected studies for full-text review. The final assessment covered 19 trials within 13 nations with participants aged 51 to 78. Vitamin D supplementation from dietary and pharmaceutical sources significantly increased serum 25(OH)D to optimal levels. Lower baseline serum 25(OH)D, lighter skin color, longer treatment duration, and prolonged skin exposure were all associated with a better response to vitamin D supplementation in postmenopausal women.

## 1. Introduction

Menopause marks a significant shift in vitamin D requirements. Postmenopausal women are particularly predisposed to vitamin D deficiency due to changes in body composition, increased age, race, inadequate sun exposure, lack of vitamin D dietary consumption, and adiposity [1,2,3,4,5]. It is becoming increasingly clear from the evidence that vitamin D deficiency is linked to various menopausal health conditions, such as vasomotor symptoms [6], vaginal atrophy or genitourinary syndrome of menopause [7,8,9], sexual dysfunction [10], and postmenopausal osteoporosis [11]. With the recognition of widespread vitamin D deficiency and its impact on the health and well-being of postmenopausal women, the importance of accurate vitamin D status assessment and a thorough understanding of the determinants of vitamin D supplementation in this population is becoming more widely recognized.

The large interlaboratory variations in assay methods used to measure 25(OH)D serum levels, the best measure of vitamin D status [12], have made defining vitamin D sufficiency difficult. Moreover, different patient characteristics and vitamin D inadequacy thresholds partly explain variations in optimal serum concentrations reported by clinical trials. However, the World Health Organization (WHO) [13], the Institute of Medicine (IMO) 2010 [14], the Endocrine Society Clinical Practice Guidelines [15], and the American Association of Clinical Endocrinologists [16] defined vitamin D deficiency as below 20 ng/mL and sufficiency as ≥30 ng/mL. These limits were established in response to evidence that secondary hyperparathyroidism becomes more common when serum 25(OH)D levels fall below 30 ng/mL.

Several interventional studies have revealed that a variety of factors influence vitamin D status and the effects of vitamin D supplementation. Body mass, basal serum 25(OH)D levels, and season of the year have all been found to be significant predictors of the vitamin D supplementation response and the impact on its status [17,18,19,20,21,22,23]. Additionally, genetic studies have demonstrated that the genotype affects serum vitamin D levels. However, findings on genetic diversity’s effect on the vitamin D supplementation response are limited [24,25,26,27,28].

Disparities in the ideal blood concentration, dose, and duration of vitamin D administration highlight the importance of considering the many factors influencing vitamin D status during menopause when implementing a supplementation plan. This is critical, given the high prevalence of vitamin D-related conditions in postmenopausal women. This review aimed to collect evidence on the factors that influence the response to vitamin D supplementation from dietary and pharmaceutical sources, as well as their impact on vitamin D status in postmenopausal women.

## 2. Methods

### 2.1. Study Protocol and Guidance

The International Platform of Registered Systematic Review and Meta-Analysis Protocols (INPLASY) assigned this protocol the registration number INPLASY202260116. This systematic review followed the recommended reporting items for systematic reviews and meta-analyses (PRISMA) 2020 [29]. Before conducting the actual search, the protocol and search strategy were peer-reviewed. This systematic review did not require any ethical approval.

### 2.2. Databases Searched and Search Strategy

The review team conducted an initial search for vitamin D-related systematic reviews and identified the literature relevant to the review questions. To validate and peer review the search strategy, the PRESS Checklist was used to evaluate the quality and completeness of the electronic search strategy [30]. We searched MEDLINE (via PubMed) from 1977 to 14 October 2022, Embase (via OvidSP) to 14 October 2022, Cochrane Central Register of Controlled Trials (CENTRAL) to 14 October 2022, CINHAL (EBSCO) from 1999 to 14 October 2022, Web of Science Core Collection from 1970 to 14 October 2022, and Scopus from 2009 to 14 October 2022. We also looked for ongoing trials on clinical trial registries such as ClinicalTrials.gov, the ISRCTN registry (https://www.isrctn.com, last accessed on 14 October 2022), and the EU Clinical Trials Register (https://www.clinicaltrialsregister.eu, last accessed on 14 October 2022). There were no restrictions on the publication date or language. The search terms and strategies were developed using the PICO (Patients, Intervention, Comparison, Outcome) model. The complete MEDLINE search strategy is described in Supplementary File. Search strategies were adjusted for each database searched. Each search was conducted three times, each by a different reviewer. The databases were last searched on 14 October 2022. We also included any publications brought to our attention by subject matter experts.

### 2.3. Study Selection

The identification of references was completed in two stages. First, all studies published from inception to October 2022 that assessed vitamin D status in postmenopausal women and associated factors were identified. EndNote 20.4 was used to import the identified studies, and duplicates were removed. Then, the remaining records were exported to Covidence Software for further screening and data extraction. Two reviewers independently assessed the eligibility of the title and abstract in an unblended standardized manner (M.M.H. and H.Z.H.). A record was included for full-text review if at least one reviewer coded it as potentially eligible. Irrelevant studies were omitted. Full texts were obtained for further review if a decision could not be reached based on the information provided in the abstract. Differences in data extraction were settled by referring to original articles and discussions in order to reach an agreement. The senior reviewer (H.Z.H.) made the final decision based on the established eligibility criteria. Additional studies were manually retrieved from the references cited in the critical articles chosen for evaluation.

### 2.4. Inclusion/Exclusion of Studies

All randomized, placebo-controlled, double-blind, single-blind trials conducted in humans and published in any language were eligible. Inclusion criteria were as follows: (1) postmenopausal women with physiological or iatrogenic causes, (2) administration of vitamin D, regardless of the source or dose, (3) at least one outcome of interest, including serum 25(OH)D or one of its metabolites, 1,25(OH)D, and (4) comparison of the outcome of interest between any pair of the following: other doses or forms of vitamin D supplementation or placebo. Abstracts were included if they contained enough information to allow data extraction. Cross-sectional, observational, and non-human studies, case reports, and trials with and without end-of-trial outcomes were excluded. We pilot-tested the eligibility criteria on a sample of reports (*n* = 6) to refine and clarify the criteria and ensure reproducibility in future research.

### 2.5. Strategy for Data Extraction and Synthesis

Three reviewers conducted the extraction process independently to minimize bias and error (M.M.H., H.Z.H., and A.R.A.). A systematized data extraction sheet was used to extract data from each record. All pertinent data were extracted from the records, and no additional information was obtained from the authors. Citations for each article were extracted, as well as the study’s first author, full title, publication date, study design, study aim, eligibility criteria, participant characteristics, types of interventions and comparators (including dosage forms, dose, and frequency), total number of participants, adverse outcomes, and study outcomes measures. Potential confounders in randomized clinical trials, if the trial was uncontrolled, the analysis technique or assay of serum 25(OH)D, and the attrition rate were also reported. We attempted to contact the authors of studies that were only published as abstracts in order to obtain more information about the study methodology.

### 2.6. Risk of Bias (RoB) Assessment

Two reviewers (M.M.H. and H.Z.H.) assessed the methodological quality of the included studies, and one reviewer (A.R.A.) arbitrated conflicts that were not due to assessor error using the Cochrane RoB 2.0 quality assessment. All included studies were evaluated for bias following the guidelines outlined in the Cochrane Handbook for Systematic Reviews of Interventions Version 6.3, 2022 [31]. Sequence generation, allocation concealment, the blinding of participants and personnel, the blinding of the outcome assessment, incomplete outcome data, and selective reporting were all investigated as potential areas of bias. Each item was assigned a RoB of low, unclear, or high.

## 3. Results

### 3.1. Search Results

**Figure 1:**
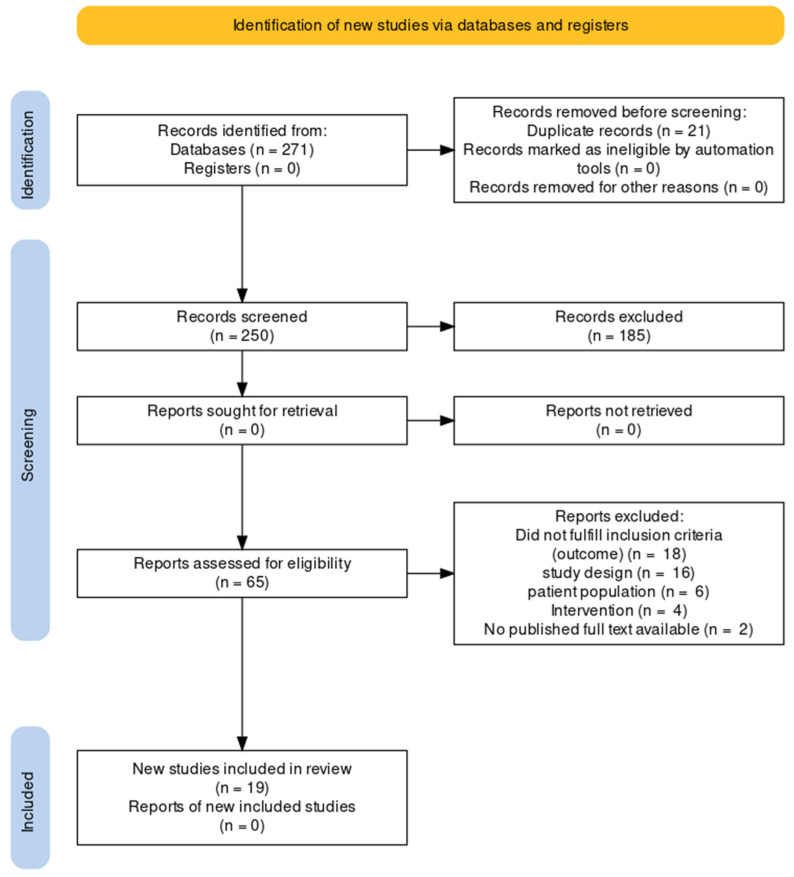
PRISMA flow diagram.

We searched MEDLINE (via PubMed), Embase (via OvidSP), Cochrane Central Register of Controlled Trials, CINHAL (EBSCO), Web of Science (WoS) Core Collection, and Scopus. The search produced 271 records. There were 21 duplicates, and the remaining 250 were screened for titles and abstracts. Covidence Software was used to screen records (titles and abstracts), review full-text articles, and extract data. During the title and abstract screening, we excluded an additional 185 records because of irrelevance to the review subject, and the remaining 65 were full-text reviewed (Figure 1). Following a search of clinical trial registries, no additional records were found. Eventually, 19 trials were included in the final review [32,33,34,35,36,37,38,39,40,41,42,43,44,45,46,47,48,49,50].

### 3.2. Study Characteristics

All 19 included studies were randomized clinical trials published in English between 2005 and October 2021. The trials included 13 countries (Thailand, Malaysia, Philippines, Indonesia, Spain, Italy, France, Greece, Switzerland, Turkey, Brazil, Argentina, and USA). The 19 trials included a total of 4677 subjects. The sample size ranged from *n* = 20 to *n* = 2077. All 19 trials were conducted on postmenopausal women who ranged in age from 51 to 78 years old. Women with osteoporosis were included in four trials [41,44,45,48]. The durations of interventions ranged from 8 weeks to 3 years. Six trials administered vitamin D as a dietary supplement (fortified milk, yogurt, or cheese) [35,37,38,39,40,46]. Other trials provided vitamin D in the form of oral supplements of vitamin D_2_, vitamin D_3_, and calcidiol. Table 1 and Table 2 summarize the key findings and participant characteristics from the studies included.

### 3.3. Risk of Bias

**Figure 2:**
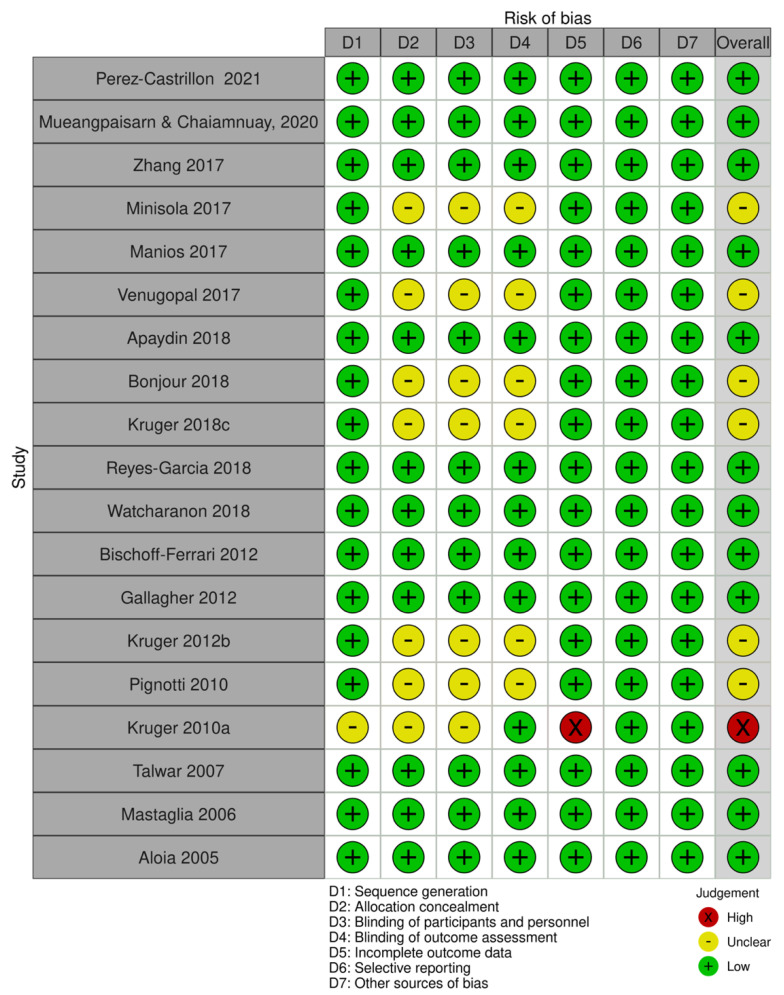
Risk-of-bias assessment [32,33,34,35,36,37,38,39,40,41,42,43,44,45,46,47,48,49,50].

The RoB assessment is detailed in Figure 2. Except for one trial [39], all trials were rated as having a low or unclear risk of bias in all aspects considered. Nine of them were double-blind to both participants and investigators [32,33,34,36,43,44,46,47,50], and one was single-blinded to participants [40]. In six trials, there was an unclear degree of selection, performance, and detection bias due to a lack of information on randomization and the concealment of the intervention allocation [35,37,38,40,45,48]. One trial had a high dropout rate (*n* = 94) for personal reasons [46]. The lack of assay standardization of serum 25(OH)D serum levels was a source of concern for all studies.

## 4. Discussion

We consider our work to be the first systematic review and a key investigation that compiled existing evidence on the effects and predictors of vitamin D supplementation on vitamin D status in postmenopausal women. Postmenopausal women are the most affected by vitamin D deficiency and the most likely to benefit from effective vitamin D supplementation. Additionally, the lack of agreement on optimal and adequate vitamin D supplementation plans in postmenopausal women has pushed this to the forefront of medical research. The findings of the 19 trials revealed that the factors linked to vitamin D status in menopausal women could be classified into two major categories: factors related to vitamin D supplementation dosage regimens, including the dose administered, frequency, formulation, and duration of treatment, and factors related to patients’ characteristics and demographics, including serum baseline, lifestyle habits, ethnicity, and genetics.

### 4.1. The Effect of Treatment Duration and Dose on Vitamin D Status

Different trials have investigated the effects of various forms and dosages of vitamin D supplementation on overall vitamin D status. A high single oral dose of 300,000 IU was found to be superior to low doses of 800 IU/day and shown to significantly increase the vitamin serum concentration [33]. Another study concluded the same with a dose of 250,000 IU of vitamin D [51]. On the other hand, Pignotti et al. concluded that low doses of 400 IU/day were ineffective at raising serum concentrations of 25(OH)D to levels considered optimal for bone health [45]. In both trials, with the administration of high doses, serum 25(OH)D returned to baseline after three months. This highlights that a maintenance dose with regular intervals would be reasonable in patients undergoing single-, large-dose vitamin D replacement. Mueangpaisarn and Chaiamnuay et al. found the same with doses of 40,000 IU and 100,000 IU of cholecalciferol after three months of treatment [43].

### 4.2. The Effects of Type of Vitamin D on Vitamin D Status

It has also been proposed that the type of vitamin D affects vitamin D status. Vitamin D_2_ was found to be effective at increasing serum 25(OH)D concentrations but required higher than the usual recommended doses of vitamin D_3_ [41]. Several studies have found that the response of serum 25(OH)D to vitamin D_2_ and vitamin D_3_ supplementation differs, with vitamin D_2_ being less effective than vitamin D_3_ [52,53,54,55]. Research attributed these differences in response between the two calciferol forms to differing affinities for the vitamin D receptor (VDR), which appear to be related to an additional step of 24-hydroxylation that inactivates calcitriol [56,57]. Moreover, it is hypothesized that vitamin D_3_ is potentially a more favorable substrate for 25-hydroxylase [58].

Bischoff et al. found that supplementation with the 25(OH)D_3_ metabolite itself is more effective and faster than vitamin D_3_ in raising 25(OH)D levels in postmenopausal women, compared to typical doses of 800 IU vitamin D_3_ [34,44]. This is because 25(OH)D_3_ is hydrophilic, has a much shorter half-life, and causes a rapid rise in serum 25(OH)D levels. Furthermore, the fact that 25(OH)D_3_ enters the circulation and bypasses first-pass metabolism makes it preferable when the fast replacement of 25(OH)D is required [59,60]. These findings are consistent with many previous studies [61,62,63,64,65,66]. Minisola et al. studied different doses of calcidiol (20, 40, and 125 ug/day) in postmenopausal women and found that all dosage regimens significantly increased serum 25(OH)D at the end of the treatment of 12 weeks; however, no difference was noticed between vitamin D-insufficient and vitamin D-deficient patients [42].

### 4.3. The Effects of Baseline Serum 25(OH)D on Vitamin D Status

Venugopal et al. found that large doses of 25,000 IU/4 weeks and 50,000 IU/4 weeks of cholecalciferol can maintain vitamin D sufficiency. However, this effect was only shown after 16 weeks of treatment in women with low baseline serum 25(OH)D receiving 25,000 IU, in contrast to women who received 50,000 IU, who started to show a rise in serum 25(OH)D at eight weeks only [48]. Similarly, after 12 weeks of treatment, patients who received standard doses of 800 IU/day and began with sufficient serum baseline 25(OH)D concentrations of 50 nmol/L were unable to achieve mean serum 25(OH)D concentrations of >75 nmol/L [67].

Additionally, Bonjour et al. and Talwar et al. emphasized the significance of baseline 25(OH)D serum concentrations and their impact on vitamin D status. They found that the lower the baseline levels, the higher the response to vitamin D supplementation, showing an inverse relationship between the two [35,47]. Other studies confirmed similar findings [19,68,69]. Many different mechanisms can explain this inverse relation. First, baseline or initial vitamin D status influences the serum 25(OH)D distribution, or the hepatic hydroxylation rate of the cholecalciferol molecule is increased by its product. Another possible mechanism is that vitamin D status could be affected by the strength of the interaction between the vitamin D molecule and its binding protein. The activity of the catabolic vitamin D 24-hydroxylase enzyme may be diminished in response to a sustained decrease in 25(OH)D serum levels [70,71,72]. These findings indicate that basal serum 25(OH)D is a significant predictor of vitamin D status, independent of the dose.

### 4.4. The Effect of Sun Exposure on Vitamin D Status

Serum 25(OH)D levels were significantly lower in patients exposed to sunlight without additional vitamin D supplementation. Sun exposure combined with vitamin D supplementation, on the other hand, resulted in a significant increase in serum 25(OH)D but only at high doses that ranged between 20,000 IU and 50,000 IU of both vitamin D_2_ and D_3_ given monthly for at least three months [48,49]. Both trials involved people of Asian descent who lived in tropical areas. For example, Wicherts et al. demonstrated that sunlight exposure had lower efficacy compared to vitamin D supplementation [73]. In addition, a six-month intervention with sunlight exposure reduced serum 25(OH)D concentrations in young women, and it was inferior to oral vitamin D supplementation or vitamin D-fortified milk in a study conducted in Saudi Arabia [74]. A meta-analysis of seven trials by Moradi et al. [75] confirmed that vitamin D_3_ significantly increased serum 25(OH)D compared to sun exposure only. Interestingly, Chel et al., on the other hand, found that UV irradiation was as effective as oral vitamin D supplementation in the elderly. However, it is worth mentioning that they used very low doses of 400 IU/day of vitamin D_3_ [76]. Furthermore, two Australian studies found that as solar UV exposure increased, serum 25(OH)D levels gradually increased, with limited sun exposure being a risk of developing a deficiency [73].

Most studies evaluating vitamin D supplementation have been conducted in countries at higher latitudes. The effect of the seasons on the response to vitamin D supplementation is well established by different studies [77,78,79]. This emphasizes the importance of supplemental vitamin D consumption during the winter season. Fortified milk with vitamin D has been demonstrated to be beneficial in boosting vitamin D status in postmenopausal women who are at increased risk due to insufficient sun exposure and calcium consumption [22,37,38,39,80,81,82]. Interestingly, Talwar et al. [47] empirically noted a pronounced peak in the serum concentration of 25(OH)D from June to September.

### 4.5. The Effect of Lifestyle Habits and Dietary Intake on Vitamin D Status

Talwar et al. and Reyes-Garcia et al. [46,47] found that age and weight-related variables were not significantly associated with changes in vitamin D serum levels. Mueangpaisarn and Chaiamnuay et al. concluded that body mass index (BMI) is not an independent factor in attaining optimal 25(OH)D levels [43]. However, one trial found that body mass index was a significant predictor of vitamin status and the dose of vitamin D_3_ [36]. Moreover, Mason et al. [83] found that weight loss through dietary restriction and exercise had no significant effect on serum vitamin D status. Lower 25(OH)D serum concentrations are associated with decreased bioavailability and increased adiposity, implying that weight loss can lead to increased 25(OH)D concentrations via decreased peripheral sequestration in a dose-dependent manner and is unrelated to changes in dietary vitamin D intake.

Manios et al. [40] examined the effects of vitamin D_3_-enriched cheese on serum 25(OH)D levels in postmenopausal women in the winter season. They found that in addition to the usual dietary intake of around 2 μg/day, a daily dose of 5.7 μg of vitamin D_3_ significantly raised serum 25(OH) D levels. Johnson et al. [84], in their partially double-blind randomized clinical trial, found that, surprisingly, the daily intake of vitamin D-enriched cheese (15 μg/day) resulted in a significant reduction in serum 25(OH)D of 6 nmol/L. In contrast, the groups that received non-enriched cheese or no cheese had either a significant increase or no change in serum 25(OH)D. These differences could be related to differences in the age of participants, the doses administered, the treatment duration, participants’ compliance with the intervention, and seasonality. Reyes-Garcia et al. [46] showed that 600 IU/day of vitamin D-enriched milk effectively raised serum levels in a large proportion of women.

### 4.6. Effect of Ethnicity and Genetics

African-American women typically require higher amounts than the recommended upper daily allowance of 2000 IU/day to achieve an optimal serum 25(OH)D concentration of 75 nmol/L, which reflects a dose of 2800 IU/day for those with concentrations >45 nmol/L and 4000 IU/day for those with serum concentrations of <45 nmol/L [32,47]. Although studies on genetic differences with regard to vitamin D supplementations are scarce, one study found that only eleven single-nucleotide polymorphisms (SNPs) were identified to be significantly linked to basal serum 25(OH)D in postmenopausal Caucasian women [50].

## 5. Limitations

The variation in the intervention strategy across all studies in terms of the vitamin D supplementation type, dose, duration, frequency of administration, and serum concentration assay methods has contributed to varying levels of heterogeneity, which may limit the ability to generalize the findings toward general practice guidelines or recommendations. However, the consistency in the results that shows the same factors that influence vitamin D status in postmenopausal women is clear from the evidence compiled from clinical trials. Furthermore, while vitamin D supplementation is important for postmenopausal women due to the effects on bone biomarkers, and while bone health is an essential and significant target for vitamin D, future research may focus on the effects of vitamin D deficiency on a broader range of postmenopausal women’s conditions, given the potential link between vitamin D deficiency and many other conditions.

## 6. Conclusions

The evidence gathered in this review on the factors influencing vitamin D status was consistent across all trials. A minimum dose of 800 IU/day and a minimum treatment duration of 12 weeks was required to reach serum 25(OH)D levels considered optimal for the health of postmenopausal women. Baseline serum 25(OH)D concentrations, below the sufficient serum baseline 25(OH)D concentrations of 50 nmol/L, were associated with a better response to vitamin D supplementation. To achieve adequate serum 25(OH)D levels, high doses of vitamin D supplementation up to 100,000 IU/week were considered safe and effective. Furthermore, a single 300,000 IU bolus dose was superior to low daily doses. Still, their inability to maintain optimal serum concentrations necessitates the regular use of maintenance doses. Vitamin D_3_ was superior to vitamin D_2_ in improving vitamin D status. Active vitamin D metabolite 25(OH)D_3_ supplementation was more effective than vitamin D_3_. Vitamin D-fortified foods can be an excellent source of increasing serum 25(OH)D, especially when sun exposure is limited. Although there is no agreement on the optimal doses or concentrations for different patient groups, clinical practice can benefit from the fact that higher doses were always preferred in specific patient groups with large safety margins.

## Figures and Tables

**Table 1:** Key findings of the included trials.

Intervention and Comparators (Dose, Frequency, and Length of Intervention)	Outcome	Baseline Mean (SD)	Outcome Mean (SD)	*p* Value	Outcome Measure	Findings
**(Pérez-Castrillón et al., 2021)** [44] **Spain and Italy**						
Calcifediol 0.226 mg/month for four months, then eight months placebo	25(OH)D_3_ (ng/mL)	12.8 (3.9)	29.7 (8.6)	<0.0001	Chemiluminescence immunoassay, the intra-assay coefficient of variation (CV) was 2.34, and the inter-assay coefficient of variation was 5.06%	Calcifediol is more effective than cholecalciferol in increasing serum 25(OH)D levels.
Cholecalciferol 25,000 IU/month for 52 weeks		13.2 (3.7)	23.8 (5.0)			
Calcifediol 0.226 mg/month for four months, then eight months placebo	Free 25(OH)D (pg/mL)	3.8 (1.1)	7.6 (2.5)			
Cholecalciferol 25,000 IU/month for 52 weeks		4.0 (1.1)	6.5 (1.6)			
**(Mueangpaisarn and Chaiamnuay, 2020)** [43] **Thailand**						
Vitamin D 40,000 IU/week for 12 weeks	25(OH)D_3_ (ng/mL)	19.4 (6.2)	34.5 (9.1)	<0.001	Electroluminescence immunoassay, the intra-assay coefficient of variability was 3.65, and the inter-assay coefficient of variability was 4.10	Higher doses were more effective at raising serum 25(OH)D compared to the low dose
Vitamin D 100,000 IU/week for 12 weeks		19.1 (6.5)	51.7 (19.3)			
**(Watcharanon et al., 2018)** [49] **Thailand**						
Vitamin D_2_ 20,000 IU/week for 12 weeks	25(OH)D_3_ (ng/mL)	29.98 (6.35)	32.44 (7.33)	<0.01	Electroluminescence immunoassay with a coefficient variation of 3.7–4.5%	A combination of both vitamin D and sun exposure is more effective in raising serum 25(OH)D levels than sun exposure only in postmenopausal women
Sunlight exposure for 12 weeks		32.30 (6.97)	29.68 (6.28)			
**(Reyes-Garcia et al., 2018)** [46] **Spain**						
Semi-skimmed milk calcium 180 mg/100 mL and vitamin D 120 IU/100 mL + Fructooligosaccharides (FOS) 5 g/L	25(OH)D_3_ (ng/mL)	21.4 (6.7)	26.6 (6.4)	<0.001	Chemiluminescence immunoassay	Daily intake of calcium and vitamin D-fortified milk significantly improves vitamin D status in postmenopausal Thai women
Semi-skimmed milk calcium 180 mg/100 mL and vitamin D 120 IU/100 mL + Fructooligosaccharides (FOS) 5 g/L		22.3 (9.4)	25.2 (6.2)			
Calcium 120 mg/100 mL and vitamin D 30 IU/100 mL		21.8 (7.1)	22.6 (7)			
**(Kruger et al., 2018)** [37] **Malaysia**						
Vitamin D 15 ug/day + calcium 1200 mg	25(OH)D_3_ (nmol/L)	62.3 (1.89)	74.8 (2.74)	<0.001	Liquid chromatography–mass spectrometry	Daily intake of calcium and vitamin D-fortified milk significantly improves vitamin D status in postmenopausal Chinese women
Regular milk powder, 428 mg calcium		64.8 (18.89)	63.1 (2.87)			
**(Bonjour et al., 2018)** [35] **Brazil**						
Vitamin D_3_-fortified yogurt 5 ug/day for 16 weeks, followed by eight weeks without product	25(OH)D_3_ (nmol/L)	36.5 (14.6)	52.6 (17)	0.008	Enzyme-linked immunosorbent assay (ELISA) and electroluminescence immunoassay. The intra-assay and inter-assay variations were less than 7% for both.	A dose-dependent improvement in serum 25(OH)D with fortified yogurt and an inversely baseline-dependent increase in serum 25(OH)D
Vitamin D_3_-fortified yogurt 10 ug/day for 16 weeks, followed by eight weeks without product		35.9 (14.8)	58.9 (19.9)	0.0008		
Regular dietary habits		36.4 (15.8)	49.5 (18.8)			
**(Apaydin et al., 2018)** [33] **Turkey**						
Vitamin D_3_ 800 IU/day for 12 weeks	25(OH)D_3_ (nmol/L)	24.2 (10.9)	57.6 (11.7)	<0.001	Chemiluminescence immunoassay	A single high oral dose of vitamin D is more effective than a daily low dose in raising serum 25(OH)D
Oral vitamin D_3_ single dose 300,000 IU		25.4 (10.9)	49.4 (17.9)			
**(Venugopal et al., 2017)** [48] **Malaysia**						
Oral cholecalciferol 50,000 IU/monthly for 12 weeks	25(OH)D_3_ (nmol/L)	90.2 (23.1)	96 (24.1)	0.057	Electro-chemiluminescence immunoassay (ECLIA). Intra-assay coefficient of variation, mean of 38.8 nmol/L −12.2% and mean of 169.5 nmol/L −2.2%	Both doses can safely maintain vitamin D sufficiency. Higher doses were required with baseline serum levels of <75 nmol/L
Oral cholecalciferol 25,000 IU/monthly for 12 weeks		91.2 (24.6)	107.1 (22.7)			
**(Minisola et al., 2017)** [42] **Italy**						
Oral calcidiol 20 ug/day for 12 weeks	25(OH)D_3_ (nmol/L)	15.1 (7.4)	49.3 (19.5)	<0.0001	Liquid chromatography–mass-spectrometry. Intra-assay coefficient variation <5% and intra-assay <6%	Calcidiol in all dosage schemes significantly increased serum 25(OH)D in postmenopausal women and can be considered an alternative to cholecalciferol
Oral calcidiol 40 ug/day for 12 weeks		16.8 (6.6)	74.8 (22.5)			
Oral calcidiol 125 ug/week for 12 weeks		16.4 (9.7)	46.4 (15)			
**(Manios et al., 2017)** [40] **Greece**						
Vitamin D_3_-enriched Gouda-type cheese 5.7 ug/day for eight weeks	25(OH)D_3_ (nmol/L)	47.3 (15.2)	52.5 (12)	<0.001	Chemiluminescence immunoassay. Intra-assay 8.9% and inter-assay 12.8%	Supplementation was sufficient in raising serum 23(OH)D levels throughout the winter season
Non-enriched reduced-fat cheese for eight weeks		42.9 (17.7)	38.3 (18.9)			
**(Zhang et al., 2017)** [50] **China**						
Cholecalciferol 1100 IU/day + calcium 1500 mg/day for 52 weeks	25(OH)D_3_ (nmol/L) reported as changes in mean 25(OH)D	74.1 (18.5)	24.31 (17.02)	<0.0001	Radioimmunoassay	Polymorphisms in CYP2R1 and GC genes may be associated with differences in response to supplements in postmenopausal Caucasian women.
Calcium 1500 mg/day for 52 weeks		73.4 (21.6)	−1.02 (11.12)			
Cholecalciferol 2000 IU/day + calcium 1500 mg/day for 52 weeks		80.1 (25.5)	31.92 (43.96)			
Control placebo		80.8 (31.7)	0.36 (41.17)			
**(Gallagher et al., 2012)** [36] **USA**						
Vitamin D_3_ for 52 weeks						
400 IU/day	25(OH)D_3_ (nmol/L)	37.8 (10.8)	Serum concentrations were reported as a dose-response mixed-effect model	<0.001	Radioimmunoassay, the inter-assay variation was 10.3% for 32.5 ng/mL and 12.7% for 70 ng/mL	Low doses of vitamin D_3_ of 600 IU and 800 IU/day were effective in raising serum 25(OH)D levels to greater than 50 nmol/L in postmenopausal women
800 IU/day		39.0 (9.5)				
1600 IU/day		37.4 (10.2)				
2400 IU/day		38.2 (10.1)				
3200 IU/day		39.8 (8.2)				
4000 IU/day		37.2 (9.2)				
4800 IU/day		38.6 (9.1)				
**(Bischoff-Ferrari et al., 2012)** [34] **Switzerland**						
Vitamin D_3_ 800 IU/day For 16 weeks	25(OH)D_3_ (nmol/L)	14.18 (3.61)	30.99 (1.59)	<0.0001	Liquid chromatography coupled to tandem mass spectrometry detection (HPLC-MS/MS)	Oral supplementation with (25(OH)D_3_ metabolite) at doses of 20 ug/day is safe and resulted in a rapid and prolonged increase in 25(OH)D levels compared with vitamin D_3_
25(OH)D_3_ (HyD) 800 IU/day for16 weeks		12.28 (4.08)	69.47 (1.58)			
Vitamin D_3_ 5600 IU/week for 16 weeks	1,25(OH)_2_D (pmol/L)	38.61 (12.10)	40.50 (2.91)	0.004		
25(OH)D_3_ (HyD) 5600 IU/week for16 weeks		33.02 (13.63)	53.06 (2.76)			
**(Kruger et al., 2012) China**						
Calcium 900 mg and vitamin D-fortified milk 6.4 ug/day for 12 weeks	25(OH)D_3_ (nmol/L)	33.13 (15.5)	(33.13–39.49)	<0.001	Electroluminescence immunoassay. Intra-assay coefficient variation is 5.1%, and inter-assay is 4.8%	High-calcium vitamin D-fortified milk was effective and significantly improved vitamin D status in postmenopausal Chinese women
Powdered control rice-based drink		29.27 (12.03)	(29.27–28.21)			
**(Kruger et al., 2010)** [39]						
High-calcium vitamin D-fortified milk—9.6 ug/day for 16 weeks (Indonesia)	25(OH)D_3_ (nmol/L)	45.06 (2.01)	-	<0.001	Electroluminescence immunoassay	High-calcium vitamin D-fortified milk was effective and significantly improved vitamin D status in two groups of Southeast Asian postmenopausal women
Powdered control rice-based drink		43.33 (2.01	-			
High-calcium vitamin D-fortified milk—9.6 ug/day for 16 weeks (Philippines)		62.0 (2.87)	-			
Powdered control rice-based drink		59.23 (2.87)	-			
**(Pignotti et al., 2010)** [45] **Brazil**						
Vitamin D_3_ 400 IU/day + 600 mg calcium for 12 weeks	25(OH)D_3_ (nmol/L)	46.67 (13.97)	59.47 (17.5)	0.023	Radioimmunoassay. Inter-assay coefficient variation 12%	Supplementation with 400 IU/day was not enough to raise serum concentrations of 25(OH)D to levels considered optimal for bone turnover in postmenopausal osteoporotic women
General orientation on a healthy diet		52.87 (21.40)	58.8 (24.72)	-		
**(Talwar et al., 2007)** [47] **USA**						
Vitamin D_3_ 800 IU/day for 24 months	25(OH)D (nmol/L)	46.9 (20.6)	65.9 (22.4)	<0.0001	Radioimmunoassay. Inter-assay coefficient variation 7%	Supplementation with 2000 IU/day oral vitamin D_3_ was sufficient to raise serum 25(OH)D concentrations to >50 nmol/L in Black African American postmenopausal women
Control		43.2 (16.8)	41.6 (18.1)	-		
Vitamin D_3_ 800 IU/day for 24 months	1,25(OH)_2_D (pmol/L)	121.3 (39.2)	107.6 (33.6)	-		
Control		119.2 (39.2)	87.4 (33.6)	-		
**(Mastaglia et al., 2006)** [41] **Argentina**						
Oral drops of vitamin D_2_—5000 IU/Day for 12 weeks	25(OH)D (nmol/L)	42.0 (23.7–45.0)	7.5 (66.2–156.2)	<0.001	Radioimmunoassay. Intra-assay coefficient variation 7.6%, and inter-assay 19%	Vitamin D_2_ was effective at raising serum 25(OH)D levels to 85 nmol/L in postmenopausal osteoporotic women
Oral drops of vitamin D_2_—10,000 IU/Day for 12 weeks		32.5 (27.5–37.5)	97.7 (79.3–123.1)			
Control (placebo)		45.0 (31.2–61.2)	55.0 (72.5–68)	<0.01		
**(Aloia et al., 2005)** [32] **USA**						
Vitamin D_3_ 800 IU/day + calcium 1500 mg/day for 24 months, then 2000 IU for another 12 months	25(OH)D (ng/mL)	19.3 (8.36)	34.8	<0.001	Radioimmunoassay	Supplementation with 20 ug/day oral vitamin D_3_ was sufficient to raise serum 25(OH)D concentrations to a mean of 89.9 nmol/L in Black African American postmenopausal women
Control (placebo)		17.2 (6.64)	-	-		
Vitamin D_3_ 800 IU/day + calcium 1500 mg/day for 24 months, then 2000 IU for another 12 months	1,25(OH)_2_D (pmol/L)	46.5 (15.2)	-	-		
Control (placebo)		45.7 (15.10)	-	-		

**Table 2:** The study participant characteristics.

Trial	Population	Sample Size	Drop out (*n*)	Adverse Events	Compliance Rate	Compliance Assessment
**(Bonjour et al., 2018** [35]	Healthy postmenopausal women	140	7	-	93−100%	Completed a questionnaire regarding adherence and acceptability in a dairy. Adherence was measured by returning yogurt lids
**(Reyes-Garcia et al., 2018)** [46]	Healthy postmenopausal women	461	94	Adverse events not reported	80%	By a food frequency questionnaire at baseline, 12 months, and end of the study
**(Kruger et al., 2018)** [37]	Healthy Chinese Malaysian postmenopausal women	121	23	Adverse events not reported	86–90%	Phone calls were made to monitor milk consumption, and each subject was provided with a monthly diary to record their intake daily
**(Manios et al., 2017)** [40]	Healthy postmenopausal women	80	1	-	97.50%	Bi-weekly meetings and telephone communication. Participants were provided with a diary to record their intake daily
**(Kruger et al., 2012)** [38]	Healthy Chinese postmenopausal women	63	5	Two events; one of constipation and stomach discomfort and one of cancer (unrelated to the intervention)	98%	Phone calls were made to monitor milk consumption, and each subject was provided with a monthly diary to record their intake daily
**Kruger et al., 2010** [39]	Healthy Asian postmenopausal women	120	3	Two events of lactose intolerance and one of gastrointestinal discomfort	98–99.6%	Participants were provided with a diary to record their intake daily
**(Pérez-Castrillón et al., 2021)** [44]	White postmenopausal women with (32) and without osteoporosis (266)	303	5	Nine adverse events were reported (not specified)	-	Dietary calcium consumption at baseline, 4, 8, and 12 months was assessed using an adapted version of a validated questionnaire
**(Mueangpaisarn and Chaiamnuay, 2020)** [43]	Asian postmenopausal women with vitamin D deficiency	94	9	Three adverse events: constipation; chest pain, and one hip fracture	>80%	Did not report
**(Watcharanon et al., 2018)** [49]	Healthy Asian postmenopausal women	52	-	None	-	Did not report
**(Apaydin et al., 2018)** [33]	Healthy postmenopausal women	60	-	-	-	Did not report
**(Venugopal et al., 2017)** [48]	Chinese Malaysian postmenopausal osteoporotic women	90	-	-	-	The study intervention was administered monthly at the clinic under the direct supervision of the investigator
**(Minisola et al., 2017)** [42]	Postmenopausal women with vitamin D deficiency	87		Four events: three reported flu and one hypercalcemia possibly related to the intervention	-	Compliance was recorded at each of the study time points (not specified how)
**(Zhang et al., 2017)** [50]	Non-Hispanic White postmenopausal women	2207	-	-	-	By bottle weight. Empty and full bottles were weighed, and then the resulting subtraction was divided by the number of tablets in the bottle
**(Gallagher et al., 2012)** [36]	Healthy postmenopausal White women with vitamin D insufficiency	163	16	Three events: one hypercalciuria, one myocardial infarction, and one congestive heart failure	86–90%	By counting pills
**(Bischoff-Ferrari et al., 2012)** [34]	Healthy White postmenopausal women	20	-			By capsule count at each study visit and by measuring the serum concentration of 25(OH)D at the end of the study
**(Pignotti et al., 2010)** [45]	Caucasian postmenopausal osteoporotic women	64	6	-	-	Dietary intake was collected through 3-day food record and checked and calculated by nutrition software (Nutrition Data System for Research, University of Minnesota)
**(Talwar et al., 2007)** [47]	Healthy Black African-American postmenopausal women	208	-	Eight severe hypercalcemia events, none of which was considered to be related to the study intervention	87%	Food frequency questionnaire at each visit to assess calcium intake
**(Mastaglia et al., 2006)** [41]	Postmenopausal osteoporotic women	45	45/7	Three cases of hypercalciuria	89–92%	By pill counts and drop counts in each box and vial returned at each monthly visit
**(Aloia et al., 2005)** [32]	Healthy Black African-American postmenopausal women	208	19	222 adverse events were reported over the study period of three years	-	Food frequency questionnaire at each visit to assess calcium intake

## Data Availability

The datasets generated during and/or analyzed during the current study will be available upon request from (Mohammed M. Hassanein, email: s2163718@siswa.um.edu.my). Data will be available for 1 year from the date the study has ended by email.

## Supplementary Materials

The following supporting information can be downloaded at: https://www.mdpi.com/article/10.3390/nu15030685/s1, Supplementary File.
