# Supplementary material for: Determinants and Effects of Vitamin D Supplementation in Postmenopausal Women: A Systematic Review
Source: Nutrients. 2023 Jan 29;15(3):685. doi: 10.3390/nu15030685 (PMC9919965; doi:10.3390/nu15030685)
Supplement: Supplementary file 1 [file nutrients-15-00685-s001.zip › nutrients-2153867-supplementary.pdf]

Supplementary file. Review Question and Complete Search Strategy of PubMed (MEDLINE)

Review Question

PICO: What are the factors that affect Vitamin D status in post-menopausal women?

Patient/ Population	Post-menopausal women
Intervention	Vitamin D Supplementation
Comparison	NIL
Outcome	Vitamin D Status/ 25(OH)D, 1,25 (OH)D

Search Strategy

A. <u>Keywords or free-text words</u>

#1	Menopause	Menopaus*[tw]OR postmenopaus*[tw]
#2	Vitamin D	"vitamin D"[tw] OR Calcifediol[tw] OR Cholecalciferol[tw]
#3	Metabolism	Vitamin D Status*"[tw] OR "serum 25-hydroxyvitamin D level*"[tw]

B. <u>MeSH Terms</u>

#1	Menopause	"Menopause"[Mesh] OR"Postmenopause"[Mesh]
#2	Vitamin D	"Vitamin D"[Mesh] OR "Calcifediol"[Mesh] OR "Cholecalciferol"[Mesh] OR "Ergocalciferols"[Mesh] OR "Hydroxycholecalciferols"[Mesh]
#3	Metabolism	"Vitamin D Response Element"[Mesh] OR "Dose-Response Relationship, Drug"[Mesh] OR "Pharmacokinetics"[Mesh] OR "25-Hydroxyvitamin D 2"[Mesh] OR "Vitamin D-Binding Protein"[Mesh]

Search Details

#**1 Postmenopause (total records = 14,114)**

("menopaus*"[Text Word] OR "postmenopaus*"[Text Word] OR "Menopause"[MeSH Terms] OR "Postmenopause"[MeSH Terms]) AND (clinicaltrial[Filter] OR randomizedcontrolledtrial[Filter])

#2 Vitamin D (total records = 6,533)

("vitamin d"[Text Word] OR "Calcifediol"[Text Word] OR "Cholecalciferol"[Text Word] OR "vitamin d"[MeSH Terms] OR "Calcifediol"[MeSH Terms] OR "Cholecalciferol"[MeSH Terms] OR "Ergocalciferols"[MeSH Terms] OR "Hydroxycholecalciferols"[MeSH Terms]) AND (clinicaltrial[Filter] OR randomizedcontrolledtrial[Filter])

#3 Dose Response (total records = 67,516)

("vitamin d status*"[Text Word] OR "serum 25 hydroxyvitamin d level*"[Text Word] OR "Vitamin D Response Element"[MeSH Terms] OR "dose response relationship, drug"[MeSH Terms] OR "Pharmacokinetics"[MeSH Terms] OR "25-Hydroxyvitamin D 2"[MeSH Terms] OR "Vitamin D-Binding Protein"[MeSH Terms]) AND (clinicaltrial[Filter] OR randomizedcontrolledtrial[Filter])

#**1 AND #2 AND #3 (total records = 148)**

(("menopaus*"[Text Word] OR "postmenopaus*"[Text Word] OR "Menopause"[MeSH Terms] OR "Postmenopause"[MeSH Terms]) AND ("clinical trial"[Publication Type] OR "randomized controlled trial"[Publication Type]) AND (("vitamin d"[Text Word] OR "Calcifediol"[Text Word] OR "Cholecalciferol"[Text Word] OR "vitamin d"[MeSH Terms] OR "Calcifediol"[MeSH Terms] OR "Cholecalciferol"[MeSH Terms] OR "Ergocalciferols"[MeSH Terms] OR "Hydroxycholecalciferols"[MeSH Terms]) AND ("clinical trial"[Publication Type] OR "randomized controlled trial"[Publication Type])) AND (("vitamin d status*"[Text Word] OR "serum 25 hydroxyvitamin d level*"[Text Word] OR "Vitamin D Response Element"[MeSH Terms] OR "dose response relationship, drug"[MeSH Terms] OR "Pharmacokinetics"[MeSH Terms] OR "25-Hydroxyvitamin D 2"[MeSH Terms] OR "Vitamin D-Binding Protein"[MeSH Terms]) AND ("clinical trial"[Publication Type] OR "randomized controlled trial"[Publication Type]))) AND (clinicaltrial[Filter] OR randomizedcontrolledtrial[Filter])
